# Neoadjuvant Immunotherapy Followed by Surgery Compared with Upfront Surgery Alone in Operable Colon Cancer with Deficient Mismatch Repair: Modeling Oncological Outcomes and Numbers Needed to Treat

**DOI:** 10.1245/s10434-024-16755-y

**Published:** 2024-12-30

**Authors:** Arezo Kanani, Torhild Veen, Dordi Lea, Claudia Zaharia, Martin Watson, Marina Alexeeva, Kenneth Thorsen, Kjetil Søreide

**Affiliations:** 1 Department of Gastrointestinal Surgery, Stavanger University Hospital, Stavanger, Norway; 2 Gastrointestinal Translational Research Unit, Molecular Laboratory, Stavanger University Hospital, Stavanger, Norway; 3 Department of Clinical Medicine, University of Bergen, Bergen, Norway; 4 Department of Pathology, Stavanger University Hospital, Stavanger, Norway

## Abstract

**Background:**

Trials on neoadjuvant immunotherapy in operable colon cancer with deficient mismatch repair (dMMR) reported high pathological response rates in the surgical specimen, but long-term survival is not known. Neoadjuvant immunotherapy as a stand-alone therapy without subsequent radical surgery is currently not investigated in colon cancer.

**Objective:**

The aim of this study was to model outcomes between trial data and real-world patients after surgery.

**Methods:**

We conducted a comparative modeling study between prospective trial data (NICHE-1) and a prospective, population-derived, translational cohort study (ACROBATICC) of patients with operable colon cancer and microsatellite instability (MSI) status. Comparison was performed across immune-cell infiltrates, stages, MSI, and patient demographics for adverse events, reported oncological outcomes, and modeling numbers needed to treat (NNT) to prevent recurrence.

**Results:**

Patient characteristics between the dMMR tumors in the NICHE-1 (*n* = 21) and ACROBATICC (*n* = 43) cohorts differed, with older patients and fewer hereditary dMMRs in the ‘real-life’ ACROBATICC cohort. Higher expression of CD8+ in dMMR tumors compared with proficient mismatch repair (pMMR) tumors was statistically significant across both cohorts. At long-term follow-up, 2/43 patients with dMMR had died from recurrent colon cancer in the ACROBATICC cohort. Assuming a curative effect of neoadjuvant immunotherapy in addition to surgery in dMMR tumors, the NNT would be >20 patients for any additional survivor.

**Conclusion:**

In unselected patients with colon cancer having dMMR, recurrence risk is very low after surgery. Assuming a curative effect of neoadjuvant immunotherapy beyond surgery alone, the NNT would be at least 20 patients to prevent one cancer death over surgery alone.

**Supplementary Information:**

The online version contains supplementary material available at 10.1245/s10434-024-16755-y.

Colorectal cancer (CRC) is a leading cause of cancer burden and deaths worldwide, and its incidence is increasing.^1^ Despite undergoing surgical treatment with intent to cure, a considerable number of patients will still develop recurrence or distant metastasis and eventually die from the disease. Hence, systemic control is important and is achieved through both adjuvant chemotherapy and, more recently, neoadjuvant therapy prior to surgery.^2,3^ In operable stages I–III colon cancer, the risk for overtreatment with adjuvant chemotherapy is of concern, as many patients can be cured from surgery alone, and the risk for recurrence may be very low.^4,5^ Unfortunately, conventional chemotherapy administered as adjuvant or neoadjuvant treatment has limitations in its effectiveness and precision; hence, a more targeted therapy is desired, preferably tailored to the intrinsic tumor biology. Immunotherapy has proven to be highly effective in patients with mismatch repair-deficient (dMMR) metastatic CRCs.^6–8^ Consequently, studies have investigated, and reported on, the use of neoadjuvant immunotherapy in resectable, early-stage colon cancer,^9^ but with no data on the effect of hard oncological endpoints such as implications on recurrence or risk of death from cancer.^10^ Furthermore, in rectal cancer with dMMR tumor, the option for an organ-sparing approach is attractive to patients,^11^ but organ-sparing may be less relevant in colon cancers. Currently, many trials are underway to further test the neoadjuvant immunotherapy approach in patients with resectable, non-metastatic colon and rectal tumors.^12^ Notably, the prevalence of dMMR in rectal cancer is <3%,^13^ while the majority of dMMR tumors are found in the colon, with a prevalence of up to 15–25% reported.^14,15^ The overall prognosis for stage I–III colon cancer is generally good, and has been reported to be excellent for patients with dMMR; however, the risk of recurrence is related to T stage and metastasis to lymph nodes.^5,14,16^

Current questions regarding the role of neoadjuvant immunotherapy in resectable colon cancer include determining which patients should receive immune checkpoint inhibitors upfront and how immune response may be elicited in early-stage colon cancers. Furthermore, even if complete response is reported at the time of pathology, the question remains whether this is sustainable over time if the tumor field is left in situ (i.e. not removed by surgery but subjected to surveillance), and if patients are still at risk of recurrence even if an initial immunotherapy effect is solicited. Another current issue is the cost of incorporating immunotherapy, as routinely administering it to all eligible patients would increase healthcare expenditure without demonstrating proven benefits to key oncological outcomes, such as the effect on overall survival.

The aim of this study was to compare data from a landmark neoadjuvant immunotherapy trial with a consecutive, real-life cohort, in terms of patient selection and putative clinical outcomes related to a scenario for which neoadjuvant immunotherapy could have been implemented. The related risk for recurrence, survival, complication patterns, and numbers needed to treat (NNT) is estimated and discussed.

## Methods

### Ethics

The Assessment of Clinically Related Outcomes and Biomarker Analysis for Translational Integration in Colorectal Cancer (ACROBATICC^17^) trial, is a study (ClinicalTrials.gov identifier: NCT01762813) conducted in accordance with national regulations and approved by the Norwegian Regional Ethics Committee (REK Helse Vest, #2012/742). Written informed consent was obtained from all participants prior to inclusion. Permission to reproduce aggregated data presented in figures from the NICHE-1 study^18^ was obtained from the publisher.

### Study Design

The current study is a modeling comparative analysis based on a phase I/II trial (NICHE-1)^18^ and a prospective, consecutive cohort (ACROBATICC).^17,19^ Results from these studies have been previously published, retrieved, aggregated, and presented for comparison. For comparative means, data have been used from both the prospective NICHE-1 trial and the ACROBATICC cohort.

### The ACROBATICC Cohort

The ACROBATICC study is an ongoing, prospective, observational, cohort study reported according to the Strengthening the Reporting of Observational Studies in Epidemiology (STROBE)^20^ recommendations, where applicable. All consecutive patients with stage I–III CRC operated with curative intent are included after providing informed and written consent. All patients were diagnosed, managed, and followed up at Stavanger University Hospital (SUH), a publicly funded university hospital within the one-payer universal health care system of Norway. The protocol^17^ and parts of the study cohort have been described in detail elsewhere.^19,21^ The current study is based on patients with stage I–III colon cancer from the initial cohort recruited between January 2013 and May 2014^19^ who did not undergo any neoadjuvant treatment before surgical resection. Follow-up data were last updated in December 2023, allowing for up to 10 years of surveillance after surgery.

### The NICHE Cohort

In the exploratory NICHE-1 study (ClinicalTrials.gov identifier: NCT03026140), patients with clinical stage I–III dMMR or pMMR colon cancer received neoadjuvant immunotherapy prior to surgery, consisting of a single dose of ipilimumab and two doses of nivolumab before surgery. Patients in the pMMR group also received celecoxib or no celecoxib. Initially, 40 patients were included in the study—21 with dMMR tumors and 20 with pMMR tumors, with one patient having both. All 40 patients underwent scheduled surgery within 6 weeks, as the primary endpoint of being safe to undergo surgery. However, five patients were excluded as two did not meet the inclusion criteria and three received nivolumab as monotherapy during the safety run-in period. Hence, 35/40 patients received dual immune checkpoint inhibition (ICI) treatment, of whom 20 were included in the dMMR group and 15 were included in the pMMR group.

In the dMMR group, 20/20 patients had a pathological response and 19/20 had a major pathological response (MPR, i.e. <10% residual tumor in the surgical specimen), while in the pMMR group, 4/15 had a pathological response and 3/4 had an MPR.^18^ In the larger phase II follow-up study (NICHE-2), 115 patients with only dMMR colon cancer were included for dual ICI treatment. Pathological response was assessed in 111 patients, of whom 109/111 had a pathological response and 105/111 had an MPR.^22^

### Histopathology, Digital Image Analysis, and Analysis of Microsatellite Instability

These analyses have been performed by board-certified pathologists via previously described methodologies.^23^ The same analyses are used in this article; however, there is a difference in the methodology for microsatellite instability (MSI) status between the NICHE-1 and ACROBATICC studies.

In the NICHE-1 study mismatch repair (MMR) status was assessed using immunohistochemistry to detect the MMR proteins MLH1, PMS2, MSH2 and MSH6, to classify tumors as MMR-deficient or -proficent.^18^

In the ACROBATICC cohort, MSI status was tested using polymerase chain reaction (PCR) and detection of changes in DNA by testing for the presence of BAT-25, BAT-26, NR-21, NR-24 and NR-27.^15,23^ The presence of two or more of these five primers classified the tumor as MSI.^23^ While we acknowledge the differences in testing, for comparison purposes, the dMMR and pMMR terminology is also used for MSI and MSS in ACROBATICC.

Furthermore, examination of CD3+, CD8+, programmed death-1 (PD-1), and programmed death-ligand 1 (PD-L1) was conducted by formalin-fixed paraffin-embedded (FFPE) immunohistochemistry in both NICHE-1 and ACROBATICC samples. In the ACROBATICC cohort, the levels of different T cells are reported for both the tumor center and the invasive margin. The same was done in the NICHE-1 study, however it was not specified whether boxplots represented levels from the tumor center or the invasive front.^18,23^ In our analyses, we compared the levels of CD3+ and CD8+ from NICHE-1 with those in the tumor center in ACCROBATICC.

Investigation of PD-L1 expression is not part of a standard panel in CRC nor used to determine eligibility for immunotherapy drugs; however, the quantification of PD-L1 expression by immunohistochemistry was performed, as previously reported,^24^ to align expression data across cohorts. Examination for dual expression of CD8+ and PD-L1 have not been conducted in the ACROBATICC study, as done in the NICHE-1 study; however, expression of CD8+ and PD-L1 was determined separately for each patient and subsequent selection of patients expressing both markers was performed in order to compare with data in the NICHE-1 cohort. In the NICHE-1 study, cut-off levels used for classifying a positive expression of CD8+ and PD-L1 are not given. In the ACROBATICC cohort, differentiation between low and high expression of CD8+ was performed, with the cut-off being the ≥75th percentile. Regarding PD-L1, tumors are classified as PD-L1 positive if >5% of the examined tumor cells expressed the ligand.^21,23^

### Adverse Events and Complications

For NICHE-1, both immune-related and surgery-related adverse events (AEs) have been reported. Surgery-related AEs were reported in both cohorts using the Clavien–Dindo classification. Immune-related AEs in NICHE-1 were classified after the Common Terminology Criteria for Adverse Events (CTCAE) version 4.0.

### Oncological Outcomes

In the NICHE-1 cohort, the oncological surrogate outcome is the pathological response in the surgical specimen. Recurrence-free survival is reported as per the updated, expanded phase I cohort in NICHE-2.

For the ACROBATICC cohort, complete long-term survival with recurrence is reported up to 10 years after surgery, and reported separately for the dMMR and pMMR groups.

### Numbers Needed to Treat

Based on the risk of recurrence in the ACROBATICC dMMR cohort and the risk of death from colon cancer, we estimated the number of patients with dMMR tumors that would be needed to treat with neoadjuvant immunotherapy in order to prevent one recurrence or one cancer-related death.

### Statistical Analysis

Statistical analysis was performed using IBM^®^ SPSS^®^ Statistics, version 29 for Windows (IBM Corporation, Armonk, NY, USA). The analysis of tumor-infiltrating lymphocytes (TILs) was conducted using an independent samples Mann–Whitney U test. The density of CD3+ and CD8+ T cells are presented in boxplots that represent the 25th percentile, median, and 75th percentile. The whiskers represent the lowest and highest data. The density of different T cells is calculated per square millimeter, similar to the reported methods in NICHE-1. The Chi-square test was used in the analysis of categorical variables, with the *p*-value calculated from the Fisher’s exact test in groups containing fewer than five events. MedCalc (https://www.medcalc.org/calc/) was used for comparison of proportions, follow-up data, and comparing complications between the two cohorts. *P*-values <0.050 were considered statistically significant and all tests were two-tailed.

## Results

**Fig. 1:**
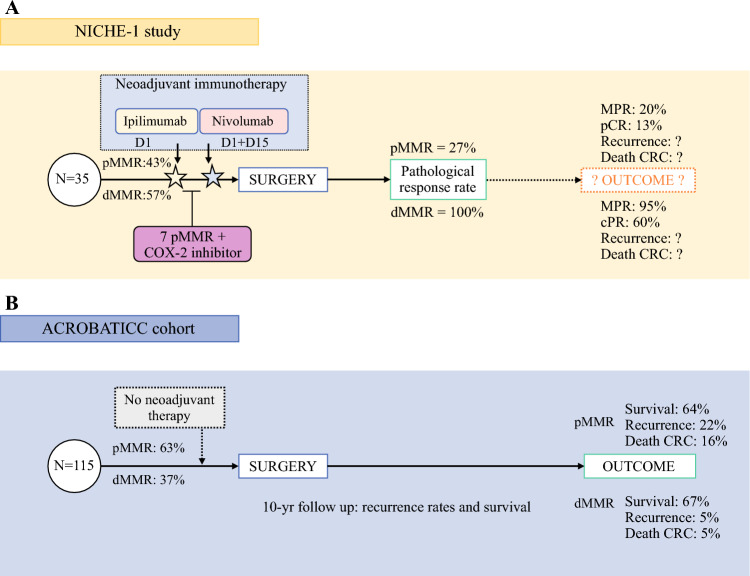
Overview of the NICHE-1 and ACROBATICC study characteristics (a simple overview of the two different studies compared in this article). **A** The NICHE-1 study included 35 patients, both pMMR and dMMR. All patients received ipilimumab on day 1 and nivolumab on days 1 and 15. Seven patients with pMMR tumors also received a COX-2 inhibitor. All patients underwent further surgery within 6 weeks. **B** The ACROBATICC study with 115 patients included as a comparative cohort without neoadjuvant immunotherapy, but with 10-year real-life follow-up and outcomes for both pMMR and dMMR. *cPR* complete pathological response, *MPR* major pathological response, *pCR* pathological complete response, *dMMR* deficient mismatch repair, *pMMR* proficient mismatch repair, *COX-2* cyclooxygenase 2, *CRC* colorectal cancer, *COX-2* cyclooxygenase 2

Comparison of patient characteristics between the two studies is presented in Table 1. In general, patients in the ACROBATICC cohort were older in both the dMMR and pMMR groups. Gender distribution reveals that the dMMR group in the ACROBATIC cohort had a higher number of females compared with the NICHE-1 group. The cohort characteristics and differences between the NICHE-1 and ACROBATICC cohorts are presented in Fig. 1.

**Table 1:** Comparing clinical characteristics between the NICHE-1 and ACROBATICC studies according to MMR status

	dMMR/MSI		pMMR/MSS		*p*-value^a^
	NICHE-1	Acrobaticc	NICHE-1	Acrobaticc	
	[*n* = 21]	[*n* = 43]	[*n* = 19]	[*n* = 72]	
Age, years [median (range)]	58.4 (22–82)	76 (50–92)	65.9 (44–77)	70 (37–91)	0.006
Sex					
Female	12 (57)	35 (81)	10 (53)	35 (49)	<0.001
Male	9 (43)	8 (19)	9 (47)	37 (51)	
Clinical stage					0.103
I	2 (9.5)	19 (44)	5 (20)	18 (25)	
II	2 (9.5)	13 (30)	7 (35)	29 (40)	
III	17 (81)	11 (26)	8 (40)	25 (35)	
Sidedness					<0.001
Right colon	14 (67)	35 (81)	8 (42)	22 (31)	
Left colon	5 (24)	2 (5)	11 (58)	44 (61)	
Transverse colon	2 (10)	6 (14)	1 (5)	6 (8)	
Lynch syndrome	7 (33)	1 (2.3)	0 (0)	0 (0)	NA

Data are expressed as *n* (%) unless otherwise specified

For the ACROBATICC cohort, further statistical analyses are performed comparing characteristics between dMMR and pMMR tumors

^a^Indicates differences between patient characteristics in the ACROBATICC cohort

*MMR* Mismatch repair, *dMMR* Deficient MMR, *pMMR* Proficient MMR, *MSI* Microsatellite instability, *MSS* Microsatellite stable, *NA* Not applicable

**Fig. 2:**
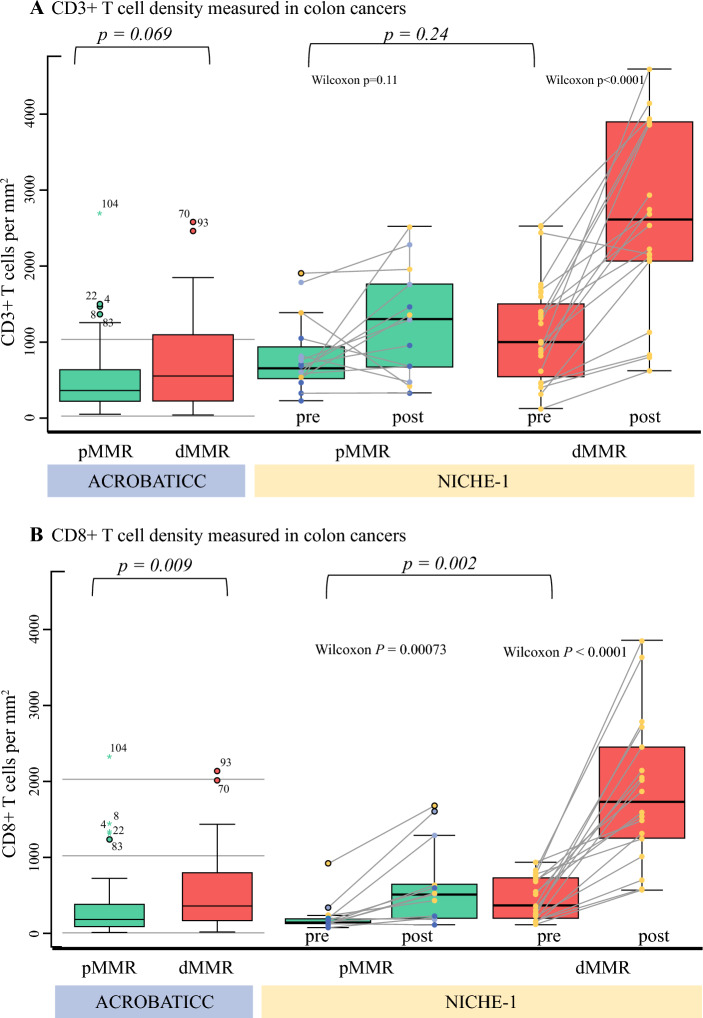
Comparison of CD3+ and CD8+ between the ACROBATICC and NICHE-1 studies. Boxplot demonstrating levels of CD3+ and CD8+ T cells in the tumor center comparing pMMR with dMMR. **A** The left boxplot is from the ACROBATICC study. There was no statistically significant difference in the CD3+ level between dMMR and pMMR in the ACROBATICC cohort (*p* = 0.069). The same findings were presented in the NICHE-1 study, which is presented in the right boxplot (*p* = 0.24). The NICHE-1 study did however show an intragroup difference in dMMR tumors before and after treatment (*p* = 0.0001). **B** The left boxplot shows a statistically significant difference in CD8+ between dMMR and pMMR (*p* = 0.009). The same results were presented in the NICHE-1 study (right boxplot; *p* = 0.002). The NICHE-1 study found a significant difference pre- and post-treatment within both pMMR and dMMR tumors (*p* < 0.0001). Graphs from the NICHE study are reproduced from Chalabi et al.,^18^ with permission from SpringerNature ^©^2020. *dMMR* deficient mismatch repair, *pMMR* proficient mismatch repair, *CD3* cluster of differentiation 3, *CD8* cluster of differentiation 3

For the dMMR group in the NICHE-1 cohort, there is an overrepresentation of patients with clinical stage III cancers, whereas clinical stage is more evenly distributed in the pMMR group. Tumor location distribution is quite similar in both cohorts. The prevalence of Lynch syndrome in the dMMR NICHE-1 cohort was higher, with one in three patients having Lynch syndrome (Table 1). Within the ACROBATICC cohort, we found a significant difference in patient age, sex, and tumor location between dMMR and pMMR tumors. The distribution of TILs for the NICHE-1 and ACROBATICC cohorts is presented in Fig. 2, with ACROBATICC data presenting the postoperative specimen evaluation.

### Follow-Up Data and Recurrence

ACROBATICC follow-up data are presented in Table 2. At up to 10 years of follow-up, both groups had a survival rate of approximately 65%. In the dMMR group, 2/43 (5%) patients had recurrence of disease. In the same group, 2/43 patients (5%) died due to CRC disease—one after recurrence of disease and the other due to postoperative complications after primary surgery. On the other hand, in the pMMR group, 16/72 (22%) had disease recurrence, whereas 12/72 (16.7%) died of colon cancer. Of the 12 patients who died of CRC disease, only two underwent surgery after recurrence.

**Table 2:** ACROBATICC long-term follow-up data after almost 10 years of follow-up

		dMMR/MSI [*n* = 43]	pMMR/MSS [*n* = 72]	*p*-Value
Status at last follow-up	Alive, NED	29 (67)	46 (64)	0.106
	Alive, with recurrence	0 (0)	0 (0)	–
	Dead, from CRC	2 (5)	12 (16.7)	0.066
	Other	12 (28)	14 (19)	0.265
Recurrence	No	41 (95)	56 (78)	0.012
	Yes	2 (5)	16 (22)	0.012
Palliative care after recurrence	No	1 (50)	8 (50)	1.000
	Yes	1 (50)	8 (50)	1.000
Cause of death	CRC	2 (5)	12 (16.7)	0.066
	Other	4 (9)	8 (11)	0.733
	Undisclosed	8 (19)	6 (8.3)	0.092

Data are expressed as *n* (%)

In the dMMR group, 67% of patients were still alive after 9–10 years of follow-up, 5% died due to CRC, and 28% died due to other causes. Similarly, in the pMMR group, fewer patients were alive at last follow-up (64%); 16.7% had died due to CRC and 19% died of other causes. The recurrence rate was 5% and 22% in patients with dMMR and pMMR tumors, respectively. In both groups, half of the patients received oncologic palliative care after recurrence of disease, while the other half received best supportive care. There was only a significant difference in recurrence of disease between dMMR and pMMR tumors.

*MMR* Mismatch repair, *dMMR* Deficient MMR, *pMMR* Proficient MMR, *MSI* Microsatellite instability, *MSS* Microsatellite stable, *NED* No evidence of disease, *CRC* Colorectal cancer

In both groups, half of the patients received palliative care after recurrence of disease—1/2 in the dMMR group and 8/16 in the pMMR group. For the NICHE-1 and NICHE-2 (only dMMR tumors) cohorts, the recurrence rate was reported to be 0%, with a median follow-up of 26 months.^22^ Recurrence or survival was not reported for pMMR patients.

In the ACROBATICC cohort, the cause of death in the dMMR group included one patient due to advanced duodenal carcinoma, one due to end-stage kidney failure, and two from cerebrovascular insults, while 8 patients did not have the specific cause of death recorded and were assumed to have died of older age-related causes as the age at the time of diagnosis was high. Among the pMMR patients, there were four other malignancy-related deaths (one each to lymphoma, metastatic melanoma, cholangiocarcinoma, and lung cancer), including one cardiac sudden death after breast cancer surgery. Two patients died from unrelated emergencies (one mesenteric bowel ischemia, one from trauma) and one from end-stage chronic obstructive pulmonary disease. Six patients had no specific cause of death and were assumed to have died of older age as there were no recoded recurrences, no new admissions to hospital, and no other work-up or treatment for other terminal illnesses (Table 3).

**Table 3:** Reported adverse events for the NICHE-1 and ACROBATICC cohorts

Complication grade	NICHE-1 [*n* = 40]		ACROBATICC [*n* = 115]
	Immune-related AEs	Surgery-related AEs	Surgery-related AEs
None	12 (30)	31 (78)	68 (59)
Grade 1–2	23 (58)	1 (2)	37 (32)
Grade 3–4	5 (13)	8 (20)	9 (8)
Grade 5	0 (0)	0 (0)	1 (<1)

Data are expressed as *n* (%)

Both the NICHE-1 and ACROBATICC complication rates were for both dMMR and pMMR tumors together. For comparison, electronic supplementary Tables 1 and 2 from NICHE-1 have been used. In the NICHE-1 cohort, all immune-related AEs have been reported, meaning more than one AE has been reported per patient, therefore the total percentage might be more than 100%. In the ACROBATICC cohort, there was no significant difference in complications between dMMR and pMMR tumors (*p* = 0.156 after calculation using the Fisher’s exact test.

*AEs* Adverse events, *MMR* Mismatch repair, *dMMR* Deficient MMR, *pMMR* Proficient MMR

### Biomarkers of Immune Response

In NICHE-1, multiple analyses have been executed to find markers between the dMMR and pMMR patient groups, and between the responder pMMR and non-responder pMMR groups. There was no statistical difference in the expression of PD-L1 between the dMMR and pMMR groups before ICI treatment. CD8+ T-cell infiltration (TCI) was found to be statistically significantly increased in both the dMMR and pMMR groups before and after ICI. Furthermore, there was a significant intergroup statistical difference in CD8+ T-cell levels pretreatment; however, there was no difference in CD3+ T cells between the two groups.

Furthermore, the only significant marker between pMMR responders and non-responders was the higher presence of CD8+/PD-L1 TCIs in the responder group. CD3+ and CD8+ T cells alone were not found to be statistically significant between the two groups. In the NICHE-1 cohort, investigators found a significant difference in the levels of CD8+ TCI, CD3+ TCI, and interferon (IFN)-γ in dMMR pre- and post-treatment tumors.

### Comparing Densities of CD3+ and CD8+ Tumor-Infiltrating T Cells

Levels of CD3+ and CD8+ in the dMMR and pMMR groups between cohorts are presented in Fig. 2. No significant difference was found in CD3+ T-cell densities in the tumor center between the dMMR and pMMR groups in the ACROBATICC cohort (Fig. 2A). Comparing the densities of CD8+ T cells between the dMMR and pMMR groups yielded significantly higher levels of CD8+ density in dMMR tumors in both cohorts (Fig. 2B), showing consistent results of higher expression in dMMR.

### CD8+ and Programmed Death-Ligand 1 (PD-L1) Expression

In the NICHE-1 cohort, dual expression of CD8+ and PD-L1 in tumor biopsies was reported to be statistically significantly higher in dMMR tumors compared with pMMR tumors.^18^ In the ACROBATICC cohort, adding these two variables together revealed that only two pMMR tumors and four dMMR tumors exhibited dual positive expression of CD8+ and PD-L1. This resulted in a non-significant difference between dMMR and pMMR tumors (*p* = 0.194).

### Numbers Needed to Treat

With close to 10 years of follow-up, only 2/43 patients in the dMMR group died of CRC. Hence, if all patients with dMMR were treated with neoadjuvant immunotherapy prior to surgery, and if immunotherapy could prevent cancer-specific deaths, one would have to treat >20 patients with immunotherapy for every (potential) added cure beyond surgery alone.

Currently, no data exist to inform whether immunotherapy as a stand-alone treatment can provide similar cure rates as surgical resection, even if high rates of complete pathological response have been reported in dMMR colon cancers.

### Complications

The NICHE-1 cohort reported immune-related grade 3–4 AEs in 5 patients (13%) and grade 3–4 surgery-related complications in 8 patients (20%) [see the ESM]. In NICHE-2, similar immune-related grade 3–4 AEs were reported in 5 patients (4%), and 12 patients (10%) reported surgery-related events, including 4 patients who experienced anastomotic leakage. However, AEs of any grade due to immunotherapy were observed in 73 patients (63%), while those related to surgery were reported in 22 patients (19%).

For the ACROBATICC cohort, surgical complications were reported for 47 patients (41%), of whom 37 patients (32%) had grade 1–2 AEs, mainly antibiotic treatment for other infections (urinary tract, pneumonia, or surgical site [skin incision] infections), gastritis, urinary retention, or transfusion due to anemia without other measures for hemorrhage. Grade 3–4 AEs occurred in 9 patients (8%). Two patients were reoperated due to anastomotic leakage, 2 were reoperated due to adhesive ileus, and 1 was reoperated due to fascial dehiscence. Furthermore, 2 patients had cardiopulmonary complications and 1 patient developed an abscess that needed radiological drainage. In the ACROBATICC cohort, one patient with grade 5 AEs died after primary surgery due to postoperative complications and multiorgan failure. The rate of complications was not significant between dMMR and pMMR tumors (*p* = 0.156) [see the ESM].

## Discussion

In this comparative study of the clinical NICHE-1 trial data and the prospective ACROBATICC cohort, we found important and relevant differences in patient characteristics between the two cohorts, likely reflecting the referral basis of the Netherlands Cancer Institute (NCI) compared with the strict population-derived Norwegian cohort. Most prominent were the higher rate of advanced-stage colon cancer (>80% stage III compared with 26%), a younger age (median age 18 years younger), and more often HNPCC (33% compared with 2%) in the Dutch cohort. Despite these clinical differences, the higher density of CD8+ in dMMR tumors seen in both cohorts suggests that the intrinsic immune biology in dMMR tumors is similar. The level of CD8+ density has also been suggested by others to be predictive of pathological complete response to immune checkpoint inhibitors,^25^ and hence may serve as a surrogate predictive biomarker.

One objective for giving neoadjuvant immunotherapy would be to prevent recurrence after surgery in dMMR colon cancers. Cancers with dMMR respond remarkably well to immunotherapy, as evaluated by pathological response,^18,22,26^ but have correspondingly poor response to adjuvant chemotherapy.^27^ Of note, dMMR frequency follows a bimodal age distribution, with the highest rates <40 years and >75 years.^27,28^ In elderly patients >75 years of age, adjuvant chemotherapy is less often administered. Cancer-specific survival may be excellent in the elderly age group as all-cause mortality is related to other diseases or higher age, rather than colon cancer per se. In the younger age group, immunotherapy may be warranted alone or as an adjunct to surgery if immunotherapy eventually demonstrates a clear influence on the disease course (beyond that of curative-intent surgery) for outcomes such as recurrence risk and overall survival in advanced-stage (T_3_-T_4_), node-positive (pN+) colon cancer with dMMR with a high risk of recurrence. Of note, due to the low event rate in these populations, large patient cohorts will need to be recruited to infer proper statistical power. Unfortunately, current recurrence models have not incorporated dMMR/MSI status as a risk factor,^4^ as MMR testing has recently become used more universally at population levels. In Norway, the recommendation of MSI testing includes only those <60 years of age, thus data on dMMR and survival in the elderly age groups are limited. Initial experience from MMR screening in CRC in Denmark found considerable variation in the use of MMR screening across population groups.^29^ Similarly, data from over 540,000 patients from the National Cancer Database found that MMR test results were only available in 22% of all patients.^27^ Hence, extrapolating results to larger patient groups, in particular the elderly, is currently hampered.

Hence, if immunotherapy is used for preventing recurrence of disease, the fiscal numbers for the NNT to prevent one recurrence would be 20 patients. This number could be reduced if high-risk groups, such as dMMR with pN+, were found to be the target group. However, in a neoadjuvant setting, this would require reliable preoperative imaging for correct cN+ staging.

A potential benefit from neoadjuvant immunotherapy, given the very high pathological response rate, could be the organ-preserving nature of the neoadjuvant approach. Currently, there are only studies on neoadjuvant immunotherapy for rectal cancer,^11^ for which surgery is postponed or deferred in favor of a watch-and-wait approach in patients with complete clinical response.^11^ However, both the location of the cancer and surgery for tumors located in the rectum are associated with a considerable different treatment and complication profile, as well as functional results and long-term quality of life after surgery, compared with that of colon cancer. Hence, the value of an organ-sparing approach for the colon may be viewed differently from that of the rectum. Also adding to the cost is the likely lifelong surveillance needed with invasive tests (i.e. colonoscopy) for monitoring of a proximal colon cancer with dMMR, as a common site for dMMR colon cancers,^14,27,30^ if treated with immunotherapy alone.

Furthermore, any effect of treatment must also be weighed against the potential for complications. For immunotherapy, immune-mediated AEs and surgical-mediated AEs must be considered. Immune-mediated grade 3–4 AEs occurred in 13% of the NICHE-1 cohort and 4% of the NICHE-2 cohort. Surgery-related grade 3–4 AEs occurred in 20% and 10% in the NICHE-1 and -2 cohorts, respectively, and were considerably lower in the ACROBATICC cohort at 8%.

Some limitations in this comparative study need to be mentioned. We are well aware of the inherent flaws in comparing the two types of cohorts, with the risk of comparing ‘apples and oranges’. However, the purpose was not to compare equal datasets but to model how any potential effect in a similar type (or community-derived) population would compare with the NICHE study, since we had access to both MSI status and intratumoral CD8+ and CD3+ expression for comparison between cohorts and long-term (>10 years) follow-up. Comparing trial data, even if not randomized, with real-world cohorts may clearly confer inherent limitations; however, it is often such study settings that are transferred to clinical practice, and hence the comparison has value for gauging the potential for clinical impact to an unselected population, such as the prospective ACROBATICC cohort. The measures for comparison should not only be viewed from the remarkable pathological response in the NICHE cohort but from the perspective of (1) Does it change the natural disease, i.e. prevent recurrence and contribute to more cure; and (2) Will it be a first step towards non-operative management if pathological response translates into durable and curable effects without surgery. As such, the study has a hypothesis-generating aim, for which future studies may be pursued.

## Conclusion

There is still a need to evaluate what role and what effect there is to expect from neoadjuvant immunotherapy to potentiate surgery or even to replace surgical resection altogether in the future. It is an attractive option to potentially replace the need for cancer surgery with that of an effective oral drug alone; however, this should only be considered if the oncological outcome is sustained over time with a comparable or favorable complication profile and with comparable oncological outcomes.

## Supplementary Information

Below is the link to the electronic supplementary material.

Supplementary file1 (DOCX 23 kb)
