# Correction: Neoadjuvant Immunotherapy Followed by Surgery Compared with Upfront Surgery Alone in Operable Colon Cancer with Deficient Mismatch Repair: Modeling Oncological Outcomes and Numbers Needed to Treat

**DOI:** 10.1245/s10434-025-16888-8

**Published:** 2025-01-09

**Authors:** Arezo Kanani, Torhild Veen, Dordi Lea, Claudia Zaharia, Martin Watson, Marina Alexeeva, Kenneth Thorsen, Kjetil Søreide

**Affiliations:** 1 Department of Gastrointestinal Surgery, Stavanger University Hospital, Stavanger, Norway; 2 Gastrointestinal Translational Research Unit, Molecular Laboratory, Stavanger University Hospital, Stavanger, Norway; 3 Department of Clinical Medicine, University of Bergen, Bergen, Norway; 4 Department of Pathology, Stavanger University Hospital, Stavanger, Norway

**Correction to: Annals of Surgical Oncology**. 10.1245/s10434-024-16755-y

In the original online version of this article the authors' given and family names were transposed.

The original article was corrected.

