# Supplementary material for: Neoadjuvant Immunotherapy Followed by Surgery Compared with Upfront Surgery Alone in Operable Colon Cancer with Deficient Mismatch Repair: Modeling Oncological Outcomes and Numbers Needed to Treat
Source: Ann Surg Oncol. 2024 Dec 30;32(5):3068–77. doi: 10.1245/s10434-024-16755-y (PMC11976776; doi:10.1245/s10434-024-16755-y)
Supplement: Supplementary file 1 — Supplementary file1 (DOCX 23 kb) [file 10434_2024_16755_MOESM1_ESM.docx]

*Supplementary information*

**Table S1: Reported adverse events for the NICHE-1 and ACROBATICC cohort**

|  | **NICHE-1**  **(n=40)** | | **ACROBATICC (n=115)** |
| --- | --- | --- | --- |
| **Complication grade** | **Immune related AE n (%)** | **Surgery related AE%** | **Surgery related AE%** |
| None | 12 (30%) | 31 (78%) | 68 (59%) |
| Grade 1-2 | 23 (58%) | 1 (2%) | 37 (32%) |
| Grade 3-4 | 5 (13%) | 8 (20%) | 9 (8%) |
| Grade 5 | 0 (0%) | 0 (0%) | 1 (<1%) |

Legend:

Both the NICHE-1 and ACROBATICC complication rate are for both dMMR and pMMR tumours together. For comparison, supplementary tables 1 and 2 from NICHE-1 have been used. In the NICHE-1 cohort all immune related AE are reported, meaning more than one AE has been reported per patient so the total percentage might be more than 100%. In the ACROBATICC cohort there was not a significant difference of complications between dMMR and pMMR tumours with P being 0,156 after calculation with Fischer exact test. Abbreviations: AE = Adverse events

**Table S2: Reported complication rate for dMMR tumours in the NICHE-2 and ACROBATICC cohorts**

|  | **NICHE-2**  **(n=115)** | | **ACROBATICC dMMR cancers(n=43)** |
| --- | --- | --- | --- |
| **Complication grade** | **Immune related AE%** | **Surgery related AE%** | **Surgery related AE%** |
| None | 42 (37%) | 93 (81%) | 20 (47%) |
| Grade 1-2 | 74 (64%) | 10 (9%) | 17 (40%) |
| Grade 3-4 | 5 (4%) | 12 (10%) | 5 (12%) |
| Grade 5 | 0 (0%) | 0 (0%) | 1 (2%) |

Legend: Comparing adverse events (AE) between NICHE-2 and ACROBATICC for only dMMR tumours. For NICHE-2 both Immune related adverse events (AE) and surgery related adverse events are reported.

For comparison, supplementary tables 3 and 4 from NICHE-2 have been used. Immune related AE are reported per incident. Hence, since some patients have experienced more than one event, percentages ass up to more than 100%.

Abbreviations: AE = adverse events

**Table S3: Comparing surgical related adverse events between dMMR and pMMR patients in the ACROBATICC cohort**

|  | **dMMR/MSI**  **(n = 43)** | **pMMR/MSS**  **(n = 72)** |
| --- | --- | --- |
| **Complication grade** | **Surgery related AE%** | **Surgery related AE%** |
| None | 20 (47%) | 48 (67%) |
| Grade 1-2 | 17 (40%) | 20 (28%) |
| Grade 3-4 | 5 (12%) | 4 (6%) |
| Grade 5 | 1 (2%) | 0 (0%) |

Legend:

Comparing surgical related adverse events between dMMR and pMMR tumours in the ACROBATICC cohort. There is no statistical difference in complication rate between the two groups with P = 0.156 using Fisher’s exact test.
